# Clinical characteristics and respiratory management of bronchopulmonary dysplasia in preterm infants: a retrospective case-control study

**DOI:** 10.3389/fped.2026.1775362

**Published:** 2026-05-20

**Authors:** Fengdan Yu, Xiaojing Xu, Mingqiong Zheng

**Affiliations:** Department of Neonatal, The First Hostpital of Tsinghua University, Beijing, China

**Keywords:** bronchopulmonary dysplasia, mechanical ventilation, neonatal diseases, pregnancy complications, preterm infants

## Abstract

**Objective:**

Bronchopulmonary dysplasia (BPD) is a common complication in preterm infants, and early recognition of clinical risk factors facilitates risk prediction.

**Methods:**

This retrospective case-control study included preterm infants admitted with BPD diagnosed and classified as mild, moderate or severe. Data were collected on perinatal characteristics, maternal factors, respiratory support, early arterial blood gas and haematologic markers and hospitalisation complications.

**Results:**

Among 410 preterm infants, 210 developed BPD and 200 did not. Infants with BPD had lower gestational age (29.36 ± 2.04 vs. 30.15 ± 1.69 weeks, *P* < 0.001) and longer hospital stays (57.65 ± 21.08 vs. 50.03 ± 17.32 days, *P* < 0.001). They required significantly longer total mechanical ventilation, including invasive and non-invasive support (349.67 ± 375.76 vs. 227.03 ± 239.33 h, *P* < 0.001), which increased with disease severity, and re-initiation of ventilation was more frequent (32.4% vs. 16.5%, *P* < 0.001). The BPD group exhibited lower base excess and higher lactate levels, accompanied by reduced haemoglobin and haematocrit (all *P* < 0.05). Maternal complications, including premature rupture of membranes, hypertensive disorders, gestational diabetes and antenatal infections, were more common in the BPD group, whose infants also showed higher rates of neonatal complications, particularly anaemia and neonatal respiratory distress syndrome. Mortality remained low but was slightly higher in the BPD group (1.5% vs. 1.0%).

**Conclusions:**

Bronchopulmonary dysplasia in preterm infants is linked to lower gestational age, adverse maternal factors and a higher rate of complications. Prolonged ventilation demonstrates a strong descriptive association reflecting disease severity and the clinical course, highlighting the need for early risk identification and individualised management.

## 1 Introduction

Bronchopulmonary dysplasia (BPD) remains one of the most common and severe chronic respiratory complications in preterm neonates, particularly among those born before 37 weeks of gestation (1, 2). Despite significant advances in neonatal intensive care, including the use of antenatal corticosteroids, surfactant therapy and non-invasive ventilation, BPD continues to contribute to substantial morbidity, prolonged hospitalisation and long-term respiratory as well as neurodevelopmental impairments (3, 4). The pathogenesis of BPD is multifactorial, encompassing arrested lung development, oxidative stress from supplemental oxygen, ventilator-induced lung injury and perinatal inflammation or infection (2, 5). Early identification of high-risk infants is essential for guiding individualised respiratory management and improving clinical outcomes (6, 7).

Previous studies have identified several perinatal and maternal factors associated with BPD. Lower gestational age is consistently linked to increased risk, and prolonged mechanical ventilation reflects a more severe clinical course, while maternal complications such as hypertensive disorders of pregnancy, gestational diabetes and premature rupture of membranes further exacerbate neonatal vulnerability (8, 9). Early laboratory parameters, including arterial blood gas values and haematologic markers, provide insight into physiological stress and oxygenation status (5). Moreover, postnatal interventions such as corticosteroid therapy, caffeine administration and recombinant human erythropoietin (rhEPO) may influence respiratory outcomes and modulate disease progression (1, 10). Collectively, these measurable factors offer critical information for evaluating BPD risk and guiding clinical management.

Despite these advances, comprehensive analyses that integrate perinatal characteristics, maternal risk factors, detailed respiratory support data, early laboratory findings and complications across BPD severity remain limited (2, 4). The present retrospective study aims to compare clinical characteristics, respiratory support parameters, early blood gas results and complications between BPD and non-BPD preterm infants. Furthermore, we investigated the relationship between mechanical ventilation duration and BPD severity to provide evidence for early risk stratification, preventive strategies and optimised management of preterm neonates at risk for BPD.

## 2 Methods and materials

### 2.1 Study design and population

This retrospective case-control study was conducted in the neonatal intensive care unit (NICU) of our hospital between January 2012 and July 2025. During the study period, standard respiratory management in our NICU prioritised early non-invasive ventilation (e.g., continuous positive airway pressure [CPAP] or non-invasive positive pressure ventilation). Invasive mechanical ventilation was initiated for infants with severe respiratory failure or those requiring intubation for surfactant administration. Weaning strategies involved a gradual reduction of peak inspiratory pressure and fraction of inspired oxygen (FiO_2_), with prompt transition to non-invasive support once spontaneous breathing efforts were adequate and blood gas parameters (e.g., partial pressure of carbon dioxide [PaCO_2_] and pH) stabilised within acceptable target ranges. Infants were included if they met all of the following conditions: (1) gestational age < 37 weeks; (2) admission to the neonatal unit during the study period with complete clinical data available; (3) parents or legal guardians voluntarily signed informed consent. Infants were excluded if they met any of the following conditions: (1) presence of major congenital anomalies, such as congenital diaphragmatic hernia or severe congenital heart disease; (2) known or suspected chromosomal abnormalities (e.g., trisomy 21 or trisomy 18); (3) diagnosed genetic or metabolic syndromes significantly affecting respiratory function; (4) incomplete medical records or missing key variables that might compromise data integrity.

Bronchopulmonary dysplasia was diagnosed according to the 2001 National Institute of Child Health and Human Development criteria (11), defined as the need for any form of supplemental oxygen (FiO_2_ > 21%) for more than 28 days after birth. Bronchopulmonary dysplasia severity was classified based on oxygen dependency and respiratory support at 36 weeks' corrected gestational age: mild BPD was defined as breathing room air without oxygen, moderate BPD as requiring supplemental oxygen with FiO_2_ < 30% and severe BPD as requiring FiO₂ ≥ 30% or the use of positive pressure ventilation, including CPAP or mechanical ventilation.

### 2.2 Data collection

Comprehensive clinical data were systematically extracted from electronic medical records using a predefined data collection form to minimise extraction bias. All data were independently verified by two investigators to ensure accuracy and completeness, and any discrepancies were resolved through discussion with a senior neonatologist.

Perinatal characteristics collected included gestational age, birth weight, sex, mode of delivery and Apgar scores at 1, 5 and 10 min. Medication exposures, including antenatal and postnatal corticosteroids, caffeine therapy and rhEPO, were also recorded. Detailed information on respiratory support was obtained, encompassing total duration of mechanical ventilation, duration of invasive and non-invasive ventilation, initial FiO_2_ and the need for re-initiation of mechanical ventilation during hospitalisation. Laboratory assessments were based on single baseline measurements rather than averages to capture the initial physiological status. Specifically, early arterial blood gas parameters (pH, partial pressure of oxygen [PaO_2_], PaCO_2_, base excess, bicarbonate and lactate) were obtained immediately prior to the initiation of respiratory support. Haematologic markers (white blood cell count, haemoglobin, haematocrit and C-reactive protein) reflect the initial sample drawn upon NICU admission (typically within the first 6 h of life).

Maternal factors documented comprised maternal age, hypertensive disorders of pregnancy, gestational diabetes mellitus, premature rupture of membranes, threatened preterm labour, placental abruption, foetal distress and antenatal or peripartum infections or fever. Finally, clinical complications occurring during hospitalisation were systematically recorded, including pneumonia, apnoea, neonatal respiratory distress syndrome (NRDS), intracranial haemorrhage, anaemia, sepsis, coagulopathy, shock, hypofibrinogenemia, feeding intolerance and respiratory failure. Clear operational definitions were applied for major complications to ensure consistency. For instance, intraventricular haemorrhage was diagnosed using cranial ultrasound and encompassed all severities (Papile grades I–IV). Pneumonia was defined by the presence of 1. high-risk factors or an acute onset; 2. respiratory system manifestations: shortness of breath, grunting, three concave signs, cyanosis, apnoea, lung rales, etc.; 3. imaging support: chest x-ray/computed tomography indicates pneumonia changes or infection indicators support: elevated inflammatory indicators + excluding other infections.

### 2.3 Statistical analysis

All statistical analyses were performed using SPSS version 27.0 (IBM Corp., Armonk, NY, USA). The normality of continuous variables was assessed using the Shapiro–Wilk test. Variables with a normal distribution are reported as means ± standard deviations, whereas those with a non-normal distribution are presented as medians with interquartile ranges. Categorical variables are summarised as frequencies and percentages. Continuous variables were analysed using t-tests or analysis of variance, and categorical variables were compared using *χ*² tests. To adjust for potential confounders and identify independent predictors of BPD, a multivariable logistic regression model was performed. Key confounders, including gestational age, birth weight, sex, antenatal corticosteroid exposure and maternal complications, were incorporated into the model. Missing data were handled using complete case analysis, as the proportion of missingness was small (<6%). Results are expressed as adjusted odds ratios (aOR) with 95% confidence intervals (CI). Additionally, to evaluate risk factors associated with BPD severity (classified as none, mild, moderate or severe), an ordinal logistic regression model was performed, adjusting for the same key confounders. Missing data for the regression models were handled using complete case analysis due to the low proportion of missingness (<6%). All statistical tests were two-sided, with *P* < 0.05 considered statistically significant.

## 3 Results

### 3.1 Comparison of baseline clinical characteristics

A total of 410 preterm infants were included in the study, with 200 infants in the non-BPD group and 210 infants in the BPD group (see Table 1 for details). Compared with the non-BPD group, infants with BPD had significantly lower gestational age (29.36 ± 2.04 vs. 30.15 ± 1.69 weeks, *P* < 0.001), while birth weight was similar between groups (1334.29 ± 517.39 vs. 1377.13 ± 328.12 g, *P* = 0.320). Maternal age was slightly higher in the non-BPD group than in the non-BPD group (32.52 ± 4.49 vs. 31.56 ± 4.56 years, *P* = 0.033). The BPD group also had a longer hospital stay (57.65 ± 21.08 vs. 50.03 ± 17.32 days, *P* < 0.001). No significant differences were observed in sex distribution, Apgar scores at 1, 5 and 10 min, mode of delivery or antenatal corticosteroid exposure. Regarding medication use, postnatal corticosteroid therapy was administered more frequently in the BPD group (54.7% vs. 42.0%, *P* = 0.010), whereas caffeine therapy was less common (88.0% vs. 97.0%, *P* = 0.001). The use of antenatal corticosteroids and rhEPO did not differ significantly.

**Table 1:** Baseline clinical characteristics between non-BPD and BPD groups.

Items	Non-BPD group	BPD group	*P* value
Number	200	210	
Sex (male: female)	111: 89	129: 81	0.223
Gestational age (weeks)	30.15 ± 1.69	29.36 ± 2.04	<0.001
Birth weight (g)	1377.13 ± 328.12	1334.29 ± 517.39	0.320
Apgar scores			
At 1 min	8.70 ± 1.56	8.52 ± 1.66	0.284
At 5 min	9.46 ± 0.73	9.32 ± 0.93	0.089
At 10 min	9.54 ± 0.63	9.51 ± 0.90	0.739
Maternal factors			
Maternal age (years)	32.52 ± 4.49	31.56 ± 4.56	0.033
Vaginal/Cesarean delivery	52: 148	60: 150	0.559
Length of hospital stay (days)	50.03 ± 17.32	57.65 ± 21.08	<0.001
Mechanical ventilation (h)	227.03 ± 239.33	349.67 ± 375.76	<0.001
Invasive mechanical ventilation (h)	66.44 ± 124.60	143.70 ± 255.77	<0.001
Non-invasive mechanical ventilation (h)	151.95 ± 159.90	205.67 ± 191.64	0.002
Initial FiO_2_	37.58 ± 13.79	41.44 ± 18.41	0.018
Medication administration			
Antenatal corticosteroid therapy	97.5%	95.3%	0.223
Postnatal corticosteroid therapy	42.0%	54.7%	0.010
Caffeine therapy	97.0%	88.0%	0.001
rhEPO therapy	30.0%	30.0%	0.995

Mechanical ventilation exposure differed markedly between the groups. Infants with BPD required longer total mechanical ventilation (349.67 ± 375.76 vs. 227.03 ± 239.33 h, *P* < 0.001), including both invasive (143.70 ± 255.77 vs. 66.44 ± 124.60 h, *P* < 0.001) and non-invasive ventilation (205.67 ± 191.64 vs. 151.95 ± 159.90 h, *P* = 0.002). Initial FiO₂ at respiratory support initiation was higher in the BPD group (41.44 ± 18.41 vs. 37.58 ± 13.79, *P* = 0.018).

Analysis of maternal risk factors showed that in the non-BPD group, the most prevalent complications were hypertensive disorders of pregnancy (38.0%), threatened preterm labour (34.0%) and premature rupture of membranes (32.5%). Gestational diabetes mellitus (24.5%), placental abruption (15.5%) and foetal distress (11.0%) were also common. In contrast, mothers of infants with BPD had higher rates of premature rupture of membranes (44.3%), hypertensive disorders of pregnancy (40.5%) and gestational diabetes mellitus (37.1%). Notably, antenatal or peripartum infection or fever occurred more frequently in the BPD group (14.8%) than in the non-BPD group.

### 3.2 Early arterial blood gas and haematologic markers

Initial arterial blood gas analysis revealed that infants with BPD had lower base excess (−3.89 ± 3.35 vs. −2.83 ± 2.81 mmol/L, *P* = 0.001) and higher lactate levels (3.08 ± 2.00 vs. 2.57 ± 1.85 mmol/L, *P* = 0.008) compared with non-BPD infants. No significant differences were observed in PH, PaO_2_, PaCO_2_ or bicarbonate levels.

Haematologic analysis showed that infants with BPD had lower haemoglobin (158.45 ± 21.94 vs. 163.07 ± 23.57 g/L, *P* = 0.040) and haematocrit (0.471 ± 0.073 vs. 0.486 ± 0.080, *P* = 0.045). White blood cell counts and C-reactive protein levels were similar between groups. See Table 2 for details.

**Table 2:** Early arterial blood gas and hematologic parameters between non-BPD and BPD groups.

Items	Non-BPD group	BPD group	*P* value
Initial arterial blood gas			
PH	7.30 ± 0.09	7.30 ± 0.11	0.567
PaO_2_ (mmHg)	81.02 ± 42.23	84.03 ± 44.29	0.485
PaCO_2_ (mmHg)	48.17 ± 11.71	49.23 ± 23.60	0.570
Base excess (mmol/L)	−2.83 ± 2.81	−3.89 ± 3.35	0.001
HCO_3_- (mmol/L)	22.22 ± 2.15	21.74 ± 2.81	0.048
Lactate (mmol/L)	2.57 ± 1.85	3.08 ± 2.00	0.008
Complete blood count			
White blood cell (10^9^/L)	9.78 ± 6.50	10.78 ± 9.68	0.223
Hemoglobin (g/L)	163.07 ± 23.57	158.45 ± 21.94	0.040
Hematocrit	0.486 ± 0.080	0.471 ± 0.073	0.045
C-reactive protein (mg/L)	4.52 ± 12.21	4.45 ± 7.94	0.943

### 3.3 Mechanical ventilation and re-initiation in relation to BPD severity

**Figure 1:**
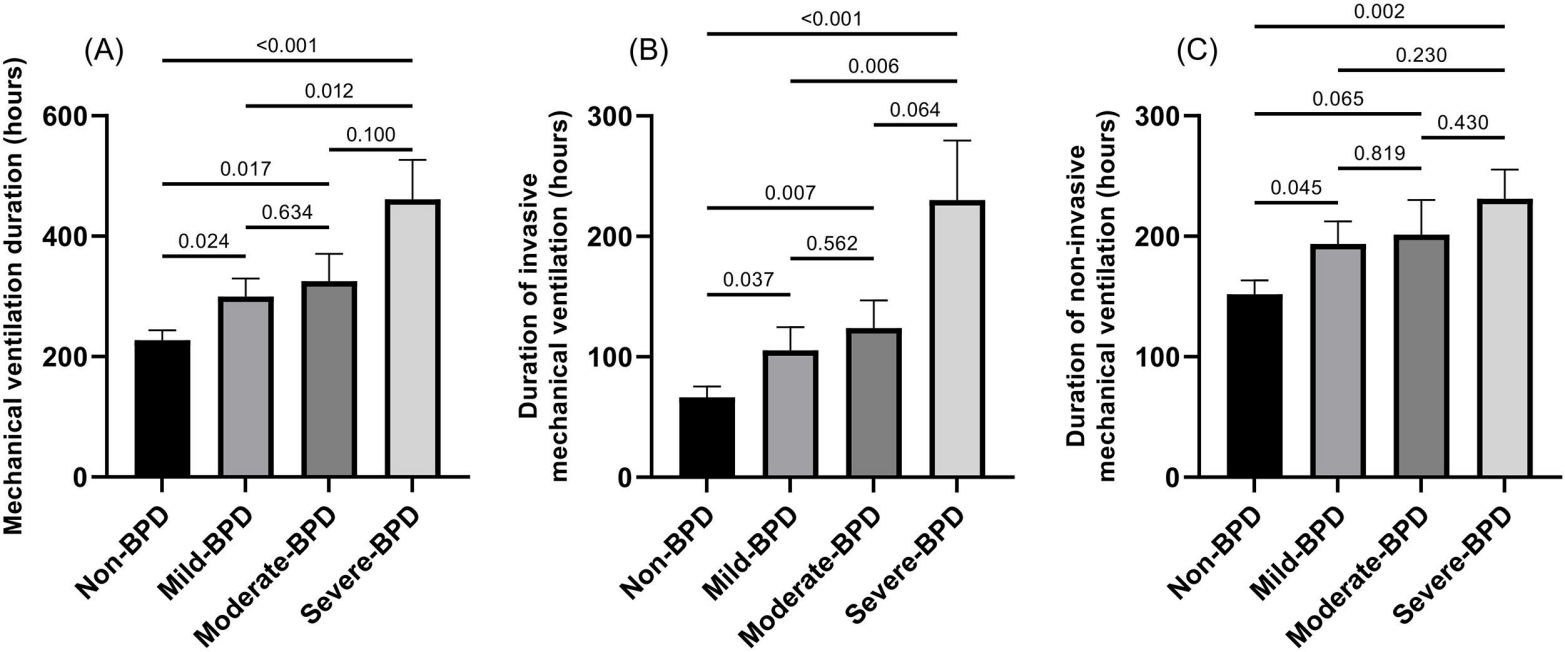
Mechanical ventilation duration in preterm infants according to BPD severity. **(A)** Mechanical ventilation duration; **(B)** Duration of invasive mechanical ventilation; **(C)** Duration of non-invasive mechanical ventilation.

Infants who developed BPD required significantly longer mechanical ventilation than those without BPD (see Figure 1 for details). Total ventilation duration increased progressively with BPD severity: non-BPD, 227.03 ± 239.33 h; mild-BPD, 299.60 ± 305.10 h; moderate-BPD, 325.20 ± 327.05 h; and severe-BPD, 461.15 ± 495.80 h.

Invasive ventilation duration also rose stepwise with BPD severity: 66.44 ± 124.60 h in non-BPD infants vs. 105.32 ± 195.51, 123.87 ± 165.09 and 230.13 ± 374.47 h for mild, moderate and severe BPD, respectively. Non-invasive ventilation followed a similar trend: 151.95 ± 159.90 h in non-BPD infants vs. 193.68 ± 189.20, 201.31 ± 205.50 and 231.03 ± 184.04 h in mild, moderate and severe BPD, respectively.

Moreover, a significantly higher proportion of infants with BPD required re-initiation of mechanical ventilation during hospitalisation (68/210, 32.4%) compared with the non-BPD group (33/200, 16.5%; *P* < 0.001), highlighting the increased respiratory support burden associated with BPD.

### 3.4 Incidence of complications and clinical outcomes

Both groups exhibited a high incidence of complications, with infants with BPD demonstrating a greater comorbidity burden. In the non-BPD group, the most frequent complications were pneumonia (92.0%), apnoea (80.0%), intracranial haemorrhage (70.0%), NRDS (60.0%), anaemia (50.0%), sepsis (50.0%), coagulopathy (45.0%), shock (40.0%), hypofibrinogenemia (40.0%) and feeding intolerance (35.0%).

Among infants with BPD, pneumonia (92.4%) and NRDS (84.8%) were most common, followed by anaemia (59.5%), intracranial haemorrhage (58.6%), shock (50.0%), sepsis (50.0%), apnoea (49.5%), coagulopathy (49.0%), respiratory failure (40.5%) and hypofibrinogenemia (37.1%). Overall, the BPD group had a higher or comparable incidence of most complications, particularly anaemia, NRDS and intracranial haemorrhage.

Regarding clinical outcomes, two infants in the non-BPD group were transferred to other hospitals, with two deaths among the remaining 198 infants (mortality rate, 1.0%). In the BPD group, five infants were transferred out, and three deaths occurred among the remaining 205 infants (mortality rate, 1.5%). Although overall mortality was low in both groups, infants with BPD showed a slightly higher mortality and an increased likelihood of requiring transfer for advanced care.

### 3.5 Multivariable analysis of risk factors for BPD

A multivariable logistic regression analysis was conducted to control for confounding effects and identify independent factors associated with BPD. After adjusting for gestational age, birth weight, sex, antenatal corticosteroid exposure and maternal complications, gestational age remained independently associated with BPD; specifically, a higher gestational age was associated with a decreased risk of BPD (aOR = 0.72, 95% CI: 0.60–0.85, *P* < 0.001). Furthermore, antenatal corticosteroid exposure was identified as a significant protective factor against BPD (aOR = 0.05, 95% CI: 0.01–0.34, *P* = 0.002). Conversely, birth weight (aOR = 1.00, *P* = 0.081), sex (aOR = 1.30, 95% CI: 0.85–1.98, *P* = 0.220) and maternal complications (aOR = 0.15, 95% CI: 0.02–1.22, *P* = 0.076) did not reach statistical significance in the adjusted model. Furthermore, an ordinal logistic regression was conducted to identify independent predictors of increasing BPD severity. Consistent with the occurrence model, higher gestational age (aOR = 0.70, 95% CI: 0.60–0.83, *P* < 0.001) and antenatal corticosteroid administration (aOR = 0.11, 95% CI: 0.03–0.35, *P* < 0.001) were significantly associated with a lower risk of progressing to a more severe BPD stage. Birth weight, sex and maternal complications were not independent predictors of disease severity (all *P* > 0.05).

## 4 Discussion

In this retrospective case-control study, we systematically evaluated clinical characteristics, maternal factors, respiratory support parameters, early arterial blood gas and haematologic markers and hospitalisation complications in preterm infants with and without BPD. Our findings confirm several established risk factors while providing additional insight into the burden of respiratory support and associated morbidities across BPD severities. Lower gestational age was a key determinant, with BPD infants significantly more premature than non-BPD infants. This aligns with prior studies emphasising the vulnerability of immature lungs to postnatal injury, including oxidative stress and ventilator-induced lung damage (1–3). Interestingly, birth weight did not differ significantly, suggesting that gestational maturity may be more critical than weight in determining susceptibility to BPD (6).

Mechanical ventilation duration demonstrated a strong descriptive association with both the incidence and severity of BPD. However, we explicitly acknowledge the inherent risk of reverse causality in this retrospective design; prolonged ventilation is likely a consequence of the clinical course and underlying pulmonary vulnerability rather than solely an independent causative factor. Total, invasive and non-invasive ventilation all increased progressively with disease severity, and the need for re-initiation of ventilation was more frequent in BPD infants. These results demonstrate a strong descriptive association between prolonged ventilatory support and BPD. Given the retrospective observational nature of this study, prolonged ventilation may reflect both an exacerbating factor for lung injury and a consequence of the underlying disease severity (10, 12, 13). Nevertheless, these observational findings are consistent with the hypothesis that minimising iatrogenic injury through early weaning strategies and non-invasive respiratory support may be beneficial, supporting previous recommendations in the literature (9, 14).

Maternal complications, including premature rupture of membranes, hypertensive disorders, gestational diabetes and antenatal infections, were more prevalent in the BPD group. These adverse intrauterine conditions may predispose preterm infants to pulmonary morbidity by promoting systemic inflammation, oxidative stress and impaired lung maturation (15, 16). For example, early low-dose hydrocortisone has been shown to improve survival without BPD in extremely preterm infants, while maternal hypertensive disorders increase the risk of severe BPD (17–19). Moreover, factors such as limited exposure to one's own mother's milk and placental complications further exacerbate respiratory vulnerability (20, 21). Recognition of maternal risk factors may therefore facilitate early risk stratification and guide individualised respiratory management in high-risk preterm populations.

Early arterial blood gas and haematologic markers differed between groups, with BPD infants exhibiting lower base excess, higher lactate levels and reduced haemoglobin and haematocrit. These findings suggest early physiological stress, impaired oxygen delivery and potential subclinical anaemia, which may exacerbate pulmonary vulnerability (5, 22). While these laboratory markers alone are not predictive and should not drive isolated clinical interventions, they complement perinatal and maternal data for early risk assessment. The spectrum and incidence of complications were higher in BPD infants, particularly NRDS, anaemia and intracranial haemorrhage. This aligns with previous cohort studies showing that BPD rarely occurs in isolation and is frequently accompanied by multisystem involvement, including haematologic, cardiovascular and neurologic complications (4, 23). Postnatal interventions also varied between groups, with corticosteroid therapy more common in BPD infants, reflecting its therapeutic use, whereas caffeine prophylaxis was less frequent, highlighting variability in respiratory management practices (1, 10).

This study has several limitations. As a single-centre retrospective analysis, unmeasured confounding factors may be present, and causal relationships cannot be established. Additionally, while we conducted multiple univariate comparisons without formal correction for multiple testing (e.g., Bonferroni) to avoid inflating Type II errors in this exploratory context, we mitigated the risk of false positives by employing multivariable regression models to confirm independent associations. Some laboratory markers were available only during the early postnatal period, limiting the ability to perform longitudinal assessments. Nevertheless, our study included a substantial cohort with detailed documentation of maternal, perinatal, respiratory and laboratory parameters, enabling a comprehensive evaluation of risk factors and disease burden (24–26). Future prospective studies incorporating longitudinal clinical, laboratory and maternal data could further refine predictive models and inform individualised interventions, ultimately contributing to reduced incidence and severity of BPD in preterm infants (27, 28), providing a foundation for improved neonatal care.

## 5 Conclusion

Bronchopulmonary dysplasia in preterm infants is closely linked to lower gestational age, prolonged mechanical ventilation, adverse maternal factors and higher rates of neonatal complications such as respiratory distress syndrome, anaemia and intracranial haemorrhage. Our findings highlight the multifactorial nature of BPD, reflecting the combined effects of immature lung development, perinatal conditions and postnatal respiratory management. Early identification of high-risk infants and individualised respiratory strategies, along with careful monitoring of clinical and laboratory parameters, are essential to mitigate disease severity. Although mortality remains low, the increased burden of complications underscores the need for proactive management. Future prospective studies integrating maternal, perinatal and early neonatal data may improve predictive models and guide tailored interventions, ultimately reducing BPD incidence and enhancing long-term outcomes in preterm populations.

## Data Availability

The original contributions presented in the study are included in the article/Supplementary Material, further inquiries can be directed to the corresponding author.
